# A Review of Eales’ Disease and *Mycobacterium tuberculosis*

**DOI:** 10.3390/biology13060460

**Published:** 2024-06-20

**Authors:** Kailey Bae, Cheldon Ann Alcantara, Jonathan Kim, Crystal Tsui, Vishwanath Venketaraman

**Affiliations:** Department of Basic Sciences, College of Osteopathic Medicine of the Pacific, Western University of Health Sciences, Pomona, CA 91766, USA

**Keywords:** Eales’ Disease, tuberculosis, idiopathic retinal periphlebitis

## Abstract

**Simple Summary:**

Eales’ Disease, an ocular condition characterized by unknown pathophysiology, exhibits a higher prevalence among young males. Since the etiology of the disease remains unclear, a comprehensive exploration of clinical presentations and diagnostic approaches is undertaken to deepen insights into its current understanding. Furthermore, potential cofactors that may trigger Eales’ Disease are reviewed, investigating the association between Eales’ Disease and other ocular conditions such as diabetic retinopathy and glaucoma. Additionally, diverse therapeutic interventions across disease stages are examined to assess the efficacy and possibility of repurposing treatments from related conditions. Through this review, the aim is to extend and elucidate potential study areas to facilitate a comprehensive understanding of Eales’ Disease and aid patients in preventing the disease or finding optimal treatment strategies.

**Abstract:**

Eales’ Disease is an idiopathic peripheral retinal vasculopathy first described by British ophthalmologist Henry Eales in 1880. Most prevalent in healthy young males, Eales’ Disease often presents with symptoms of sudden blurry or decreased vision and floaters. Although no clear, standardized stage of the disease exists, it progresses through three overlapping phases—peripheral periphlebitis, ischemic capillary ischemia, and retinal neovascularization. The etiology of Eales’ Disease is unknown and appears to be multifactorial, but post-TB hypersensitivity to tuberculoprotein and *M. tuberculosis* DNA is the most potential cause in the etiology of Eales’ Disease. With a thorough examination of the clinical presentation and diagnosis of Eales’ Disease—incorporating the latest clinical findings related to the condition—the investigation for Eales’ Disease extends to explore recent potential connections with other ocular conditions or possible cofactors, such as glaucoma, uncontrolled diabetes, drug abuse, or inherited medical conditions. Moreover, focusing on critical insights into the treatment of Eales’ Disease across its various stages of progression, the overarching goal of the paper is to refine and suggest possible future diagnostic and therapeutic strategies. Widening our understanding of pathophysiology and utilizing various treatment options for individual patients holds immense potential for advancing ocular medicine and optimizing patient care for people with this disease with unknown pathophysiology.

## 1. Introduction

Eales’ Disease was first described as idiopathic obliterative vasculopathy by the British ophthalmologist Henry Eales in 1880. This condition, marked by recurrent vitreous hemorrhages, predominantly affects young male adults and is most prevalent in regions such as India, Southeast Asia, and Germany [1]. While the precise incidence rate and etiology of Eales’ Disease is unknown, *M. tuberculosis* appears to be the leading cause of this disease, as evidenced by the high prevalence of positive tuberculosis test results among patients with Eales’ Disease, including the Mantoux screening test, PCR, and interferon-gamma release assay [2].

Although no other diseases have been definitively linked to the development of Eales’ Disease, there are intriguing overlaps with other ocular conditions, such as diabetic retinopathy and glaucoma, in terms of pathogenesis and histopathologic features, suggesting a potential area for further investigation [3].

The current clinical presentation defines three overlapping stages in the progression of retinal changes associated with Eales’ Disease, each characterized by specific clinical findings to help detect and assess the severity to guide the therapeutic approaches accordingly. Treatment during the inflammatory stage typically involves systemic steroids combined with antituberculosis therapy [4]. As the disease advances, interventions such as photocoagulation, the anti-vascular endothelial growth factor (VEGF), intravitreal injections, and vitrectomy surgery become necessary to halt disease progression. Further exploration into the association between Eales’ Disease and other ocular diseases is anticipated to broaden our understanding of the disease and refine treatment strategies.

## 2. Eales’ Disease Clinical Presentation

**Figure 1:**
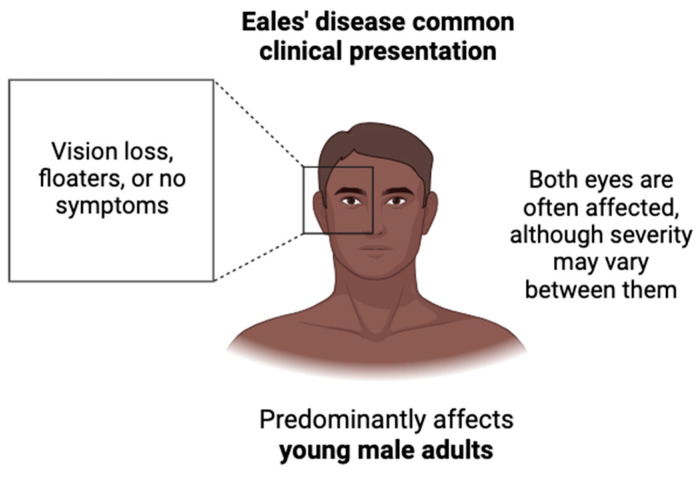
Common clinical presentation of Eales’ Disease. Eales’ Disease predominantly affects the eyes of young male adults with varying levels of severity, from asymptomatic to vision loss to increased floaters.

**Figure 2:**
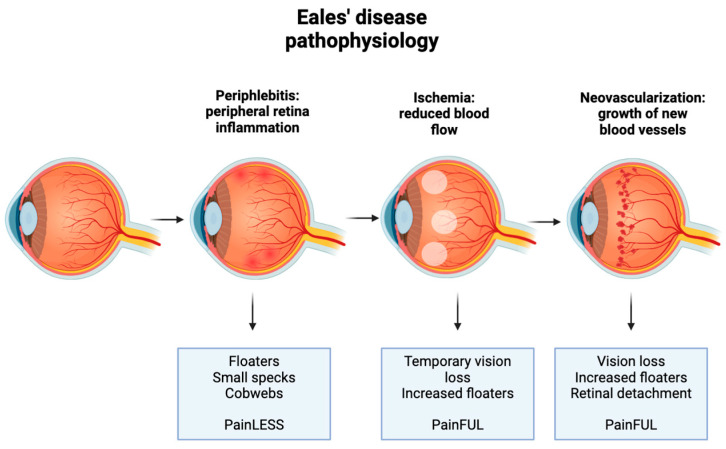
Pathophysiology of Eales’ Disease. Three stages of the disease typically occur, each with specific pathways that produce symptoms and/or pain.

The most common reason for the initial consultation of Eales’ Disease is decreased visual acuity secondary to vitreous hemorrhage [3,4,5]. Clinical presentation depends on the stage of the disease, which is categorized by the progression of retinal changes. Initial stages may be asymptomatic. Both eyes are often affected, although the severity may vary between them [5,6]. Zhao et al. found that the most common complaints from patients with Eales’ Disease were decreased vision loss, no complaints, or floaters only, as shown in Figure 1. Three stages of the disease typically occur, each with a characteristic hallmark sign, as represented below in Figure 2. The first stage manifests as retinal phlebitis or inflammation of the peripheral veins of the retina. At this stage, patients may first report floaters, small specks, or cobwebs in both eyes that can affect their vision with no accompanying pain. The second stage manifests as peripheral nonperfusion or ischemia. This lack of blood supply stimulates the next stage. The third stage manifests as retinal neovascularization, either on the optic disc or elsewhere on the retina. The new vessels are fragile and can bleed, leading to complications such as bleeding into the vitreous humor. This may cause sudden vision loss or a sudden increase in floaters. The new vessels regress spontaneously, which may cause retinal traction detachment [5]. It is important to note that not all patients with Eales’ Disease progress through all three stages, and the severity and progression of the disease can vary among individuals. Additionally, the boundaries between stages may not always be clearly defined, and there can be overlap in clinical features.

## 3. Eales’ Disease Diagnosis

There is no specific diagnostic test to confirm Eales’ Disease; therefore, it is a diagnosis of exclusion [1]. There are a few methods of determining stage severity. Fundus fluorescein angiography may demonstrate inflamed vessel walls or delayed venous filling. A B-scan ultrasound may be used to determine whether retinal detachment is present. Optical coherence tomography may be used to show macular edema [7]. Other causes of decreased visual acuity or retinal vasculitis should be ruled out. A complete blood count can rule out leukemia and other hematological diseases. Erythrocyte sedimentation rate, blood sugar, and a coagulation profile may rule out systemic inflammatory diseases, diabetic retinopathy, and blood clotting disorders, respectively. The high-resolution computed tomography chest or chest X-ray and Mantoux test may rule out tuberculosis. Hemoglobin electrophoresis can rule out sickle cell retinopathy. Patients with suspected Eales’ Disease should undergo a comprehensive examination of both eyes by an ophthalmologist. Regular follow-up and monitoring are essential to assess the progression of the disease and adjust the treatment plan accordingly.

## 4. Eales’ Disease Pathophysiology

Eales’ Disease generally presents as inflammation and ischemia with resultant neovascularization and hemorrhage [7,8]. These overlapping events occur predominantly in the peripheral retinal veins, but several case reports have reported arterial as well as macular involvement [9,10,11]. The early stage of Eales’ Disease is characterized by peripheral retinal periphlebitis with associated venous dilation and perivascular exudates [7,8]. Extensive inflammation and vasculitis can eventually lead to vascular occlusion, thus impairing the perfusion of the peripheral retina [7,12,13].

Persistent ischemia stimulates the generation of VEGF, consequently leading to neovascularization. The neovascularization observed in patients with Eales’ Disease is thought to be a misguided response to the ischemia that involves several inflammatory markers in addition to VEGF [7,14,15].

The ischemia-driven neovascularization correlates clinically with recurrent vitreous hemorrhages often seen in patients with Eales’ Disease. On fundoscopy, patients with Eales’ Disease are likely to display vascular changes, such as tortuous vessels, sclerosis, abnormal anastomoses, and inconsistent vessel caliber [7,12,13].

The precise etiology of Eales’ Disease remains unknown, though numerous studies have shed light on the possible involvement of *M. tuberculosis* and *M. fortuitum* in the likely multifactorial development of Eales’ Disease [2,16,17,18,19]. Madhavan et al. have used PCR to detect a statistically significant presence of *M. tuberculosis* DNA in the epiretinal membrane of patients with Eales’ Disease [17].

In a retrospective study of 50 patients with Eales’ Disease, patients tested for four different types of common tuberculosis tests, including the Mantoux test, QuantiFERON-TB Gold test, high-resolution computed tomography of the chest, and PCR from the anterior chamber tap or vitreous tap [2]. All patients tested positive for one or more tests, providing additional evidence to support the connection between *Mycobacterium tuberculosis* infection and Eales’ Disease [2]. However, as cultures of vitreous samples showed no growth of *M. tuberculosis* [2], this result sheds light on the role that post-TB hypersensitivity to tuberculoprotein and *M. tuberculosis* DNA play in the etiology of Eales’ Disease [2,20,21]. Further complicating the pathologic role of *M. tuberculosis*, Eales’ Disease has been observed in patients who are Mantoux-negative [22].

A recent case report of a patient diagnosed with Eales’ Disease with a history of cocaine abuse further introduced an alternative perspective on the pathophysiological route of Eales’ Disease [23]. While he tested negative on multiple tuberculosis tests, including the QuantiFERON TB-gold test, CT scan, and ACE test, he presented a family history of coagulation disorders and a 10-year history of cocaine abuse. Iannetti et al. hypothesized that cocaine abuse and coagulation disorders may serve as potential cofactors in triggering Eales’ Disease. The correlation between these cofactors and Eales’ Disease should be investigated for a comprehensive understanding of Eales’ Disease [23].

## 5. Association between Eales’ Disease and Other Ocular Conditions

Diabetic retinopathy often develops as a complication of chronic diabetes mellitus, especially uncontrolled diabetes. About a third of individuals with diabetes have signs of diabetic retinopathy [24]. Chronic exposure to conditions, such as hyperglycemia and hypertension is believed to initiate a cascade of events that lead to microvascular damage and retinal dysfunctions [24]. Different metabolic pathways have demonstrated the effects of hyperglycemia-induced vascular damage, such as the polyol pathway, advanced glycation end products (AGEs) accumulation, the protein kinase C (PKC) pathway, and the hexosamine pathway [25]. Despite multiple mechanisms, the result is cytokine and growth factor activation, leading to vascular endothelial dysfunction and including increased vascular permeability and microvascular occlusion [26]. These changes can cause microaneurysms, edema, hard exudates, and hemorrhages within the retinal vasculature [24]. Consequently, microvascular occlusions may cause retinal ischemia, which leads to neovascularization [26].

While Eales’ Disease and diabetic retinopathy have different etiologies, both conditions have similar signs of the disease, including pre-retinal neovascularization and potential blindness [3]. Nebbioso et al. found that retinal damage in various retinal pathologies can result from oxidative stress induced by reactive oxygen species and reactive nitrogen [27]. In both the pathogenesis of diabetic retinopathy and Eales’ Disease, superoxide reacts with nitric oxide, producing peroxynitrite (ONOO), a highly reactive substance that can induce cell damage [27]. Histologically, features of epiretinal membranes in Eales’ Disease are noted to be comparable to other vasoproliferative disorders, such as diabetic retinopathy, except for the presence of more inflammatory cells, as evidenced by Majji et al. [28]. Additionally, Murugeswari et al. showed that vitreous specimens from proliferative diabetic retinopathy and Eales’ Disease patients have the same elevations of pro-inflammatory and pro-angiogenic cytokines, specifically, interleukin-6 (IL-6), interleukin-8 (IL-8), monocyte chemoattractant protein-1 (MCP-1) and VEGF [3]. The overlap in pathogenesis, histopathologic features, and cytokine profiles suggests an association between these two conditions but merits further study.

Glaucoma is another ocular disease that can cause irreversible vision loss. It is defined by a group of eye conditions marked by the gradual depletion of retinal ganglion cells, often due to elevated intraocular pressure, which results from the dysregulation of the aqueous outflow pathways in the eye [29]. Glaucoma may also develop secondary to other ocular conditions that elevate intraocular pressure, thus leading to optic nerve injury. Some common causes of secondary glaucoma include ocular trauma, anterior segment neovascularization, and some systemic medications [29]. Although not well-documented, secondary neovascular glaucoma could potentially develop in individuals with advanced Eales’ Disease as a rare complication, as demonstrated in a case report by Alfayyadh et al. [30]. This patient presented with a decrease in left eye vision, eye pain, frontal headache, vitreous hemorrhage, and an increase in intraocular pressure and was found to have a positive PPD test and advanced stage of retinal ischemia, which resulted in an uncommon presentation of Eales’ Disease as neovascular glaucoma [30]. After management with anti-glaucoma, intravitreal anti-VEGF, and laser photocoagulation, the patient’s visual acuity improved [30]. This case report highlights the importance of properly diagnosing Eales’ Disease as rare presentations may be seen and warrant further investigation into the association with glaucoma.

## 6. Treatment

The objective of Eales’ Disease treatment is to reduce retinal perivasculitis and related vitritis [31]. The treatment can be broadly classified into surgical and non-surgical interventions, each potentially augmenting the efficacy of various treatments.

Corticosteroids are widely utilized treatments for Eales’ Disease, demonstrating significant effectiveness, particularly in patients experiencing the acute inflammatory stage of the condition [21,32]. Intravitreal steroids using an intravitreal triamcinolone injection showed notable improvement in idiopathic retinal vasculitis [33]. However, for individuals who do not respond well to or cannot tolerate corticosteroids, alternative therapies such as cyclosporine and azathioprine are available [21].

Laser photocoagulation serves as the primary treatment for the proliferative stage, aiming to prevent neovascularization. This treatment showed significant improvement in visual outcomes by stabilizing retinal lesions and maintaining vision [32,34]. In cases where photocoagulation is insufficient or not preferred due to other conditions, intravitreal injections of vascular endothelial growth factor (anti-VEGF) inhibitors are performed to prevent neovascularization [35].

**Figure 3:**
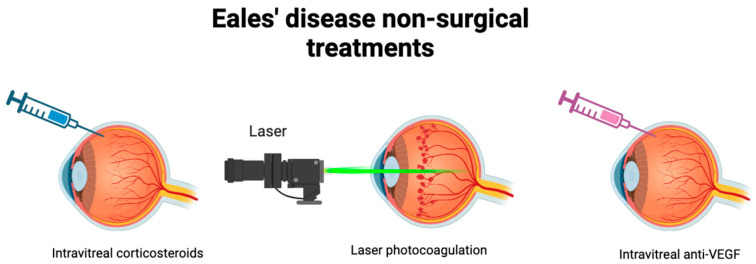
Non-surgical treatments of Eales’ Disease. Varying stages of the disease may be better targeted with certain therapies. Intravitreal corticosteroids are widely utilized to combat the acute inflammatory stage. Laser photocoagulation targets the proliferative stage to prevent neovascularization. Intravitreal anti-VEGF may also be utilized in combination to prevent neovascularization.

Anti-VEGF represents synergistic therapy often combined with previously mentioned treatments (Figure 3). A rare case involved a patient with recurrent vitreous hemorrhage unresponsive to intravitreal anti-VEGF therapy, who was effectively treated with a combined therapy of photocoagulation and anti-VEGF therapy of ischemic retinal areas, resulting in improved visual acuity without signs of recurrence [36,37,38]. Anti-VEGF therapy is globally recognized as a safe and widely utilized treatment for retinal diseases without any severe drug-related ocular events [39,40,41]. This treatment in the early stage of Eales’ Disease seems to reduce the necessity of vitrectomy and helps recovering patients with Eales’ Disease with visual loss from hyphemia [39]. However, anti-VEGF therapy for various retinal diseases could cause ocular hypertension (OHT) after the injection. Therefore, the possibility of OHT and glaucoma must be considered after the injection [42].

Vitrectomy is the final stage of treatment performed when non-resolving vitreous hemorrhages last for longer than 3 months [21]. Although typically considered a last resort, earlier vitrectomy prompted by early posterior vitreous detachment allows surgeons to achieve safe surgery with satisfactory results [21,43]. Notably, studies have demonstrated a high success rate of vitrectomy in improving the visual acuity of patients with vitreous hemorrhage or tractional retinal detachment [44]. Further investigation into the combination of anti-VEGF therapy could be beneficial in preventing disease advancement in its early stages. Early diagnosis is crucial to prevent serious complications, such as neovascular glaucoma [30].

## 7. Discussion

As the etiology of the disease remains elusive, there are numerous areas yet to be explored. While tuberculosis has been strongly implicated as a potential trigger of the disease, investigating the immunohistory of larger patient cohorts would be invaluable for a comprehensive understanding and accurate diagnosis of the disease. Additionally, other potential cofactors, such as drug abuse or inherited medical conditions, warrant thorough investigation to unravel the full pathophysiology of the disease.

In addition to conventional ocular disease treatments, involving steroids, combination therapies with photocoagulation and anti-VEGF therapy have shown efficacy in regressing disease progression, particularly in cases where initial treatments are insufficient or inaccessible. Early detection plays a pivotal role in preventing the disease from progressing to the proliferative stage, which often requires more intense interventions, such as vitrectomy and intravenous treatments. Furthermore, early detection can mitigate the development of more complex conditions, such as neovascular glaucoma [30].

Beyond tuberculosis, which has been notably linked to the etiology of Eales’ Disease, further studies on rare case reports of patients presenting with complex medical histories and potentially associated diseases are warranted. As exemplified by Mercut et al., Eales’ Disease demands a multidisciplinary approach, involving collaboration among gastroenterology, infectious diseases, pulmonology, and rheumatology departments [45]. This multidisciplinary approach, involving diverse specialties, is helpful in diagnosing and formulating precise treatment schemes, particularly for patients with challenging medical conditions, such as those with immunosuppressive requirements. By integrating expertise from immunologists and ophthalmologists, along with other relevant departments, a comprehensive understanding of the disease can be investigated, leading to the development of optimal treatment strategies.

## 8. Conclusions

Eales’ Disease presents a compelling area for exploration by scientists and clinicians, offering potential insights into its unknown aspects. With its etiology still unclear, investigating its pathophysiology and devising treatment strategies that offer improved efficacy while minimizing invasiveness is necessary. Exploring potential associations with other conditions may uncover unexpected links beyond the commonly attributed tuberculosis. Moving forward, a comprehensive understanding of the disease requires a multidisciplinary approach, drawing upon insights from diverse clinical fields besides immunology and ophthalmology. By analyzing potential associations with other diseases, treatment modalities can be optimized to enhance patient outcomes and prevent disease progression with minimal intervention.

## Data Availability

Not applicable.
